# Editorial: Women in science: pediatric pain

**DOI:** 10.3389/fpain.2025.1539856

**Published:** 2025-03-19

**Authors:** Anna M. Hood, Karen E. Weiss, Maria Forsner

**Affiliations:** ^1^ Division of Psychology and Mental Health, Manchester Centre for Health Psychology, University of Manchester, Manchester, United Kingdom; ^2^ Department of Psychiatry and Psychology, Mayo Clinic, Rochester, MN, United States; ^3^ Department of Nursing Sciences, Umeå University, Umeå, Sweden

**Keywords:** pediatric, pain, women, editorial, equity

**Editorial on the Research Topic**
Women in science: pediatric pain

The persistent underrepresentation of women in science reflects a broader systemic issue of gender inequity. Women comprise less than 30% of researchers globally (1); this disparity is rooted in longstanding biases and stereotypes that discourage girls and women from pursuing careers in science-related fields. Critically, women researchers from minoritized backgrounds are further underrepresented, facing additional systemic, institutional, and cultural barriers (2). These disparities limit the diversity of perspectives and ideas essential for addressing complex scientific questions, including pediatric pain, which requires diverse thoughts and experiences, innovative approaches and interdisciplinary collaboration. The commitment to promoting gender equity in research is vital for achieving justice and unlocking the potential of sustainable development.

By spotlighting the contributions of women researchers, this *Research Topic* aligns with UNESCO's goals of fostering gender parity in science. This *Research Topic* celebrates the contributions of women researchers in pediatric pain, showcasing their work on diverse topics ranging from neurobiological mechanisms, diagnostic uncertainty, and coproduction of research to technological advancements, friendship, and social determinants of health. By promoting this collection, we aim to elevate women's voices in science, encourage future women scientists, and address critical issues in pediatric pain management. These studies not only deepen our understanding of pediatric pain but also inspire innovative solutions to improve the lives of young people living with pain.

Our first article was the longitudinal study, “*Are They Still Friends? Friendship Stability of Adolescents With Chronic Pain: 1-Year Follow-Up,”* which revealed that same-sex friendship dyads, including adolescents with chronic pain (ACP), experience higher rates of friendship breakup compared to non-pain dyads. Chronic pain and shorter friendship duration were significant predictors of a breakup, highlighting the vulnerability of these friendships. Adolescence is a critical period where friendships play a vital role in emotional and social development. These findings advocate for strategies to foster long-term, supportive friendships for ACPs to mitigate the emotional toll of chronic pain (Forgeron et al., 2022).

The study, “*Amygdalar Functional Connectivity Differences Associated With Reduced Pain Intensity in Pediatric Peripheral Neuropathic Pain,”* delves into the neural mechanisms underlying pain in adolescents with peripheral neuropathic pain. Utilizing resting-state functional MRI, the research identifies significant differences in amygdalar connectivity with other brain regions, including the dorsolateral prefrontal cortex and angular gyrus. Notably, the study highlights sex-dependent variations in pain-related brain activity, suggesting distinct mechanisms in girls and boys. This research enhances our understanding of pediatric pain neurobiology and underscores the importance of sex-specific approaches in future studies (Verriotis et al., 2022).

Diagnostic uncertainty is a common challenge in pediatric chronic pain management, often complicating referrals to specialized programs. The Delphi study, “*Attaining Expert Consensus on Diagnostic Expectations of Primary Chronic Pain Diagnoses for Patients Referred to Interdisciplinary Pediatric Chronic Pain Programs,”* addresses this issue by achieving consensus among pain specialists on diagnostic criteria for six chronic pain diagnoses, including Complex Regional Pain Syndrome (CRPS) and chronic pelvic pain.

The study identifies key diagnostic indicators and highlights areas where agreement is strongest, such as CRPS, whilst acknowledging gaps in consensus for conditions like chronic pelvic pain. Such work will be instrumental in standardizing referral expectations, reducing unnecessary tests, and improving access to care (Greenough et al., 2022).

Pain-related stigma is a significant but underexplored social determinant of health in pediatric pain populations. The mini-review, “*Pain-Related Stigma as a Social Determinant of Health in Diverse Pediatric Pain Populations,”* introduces a conceptual framework for understanding how stigma intersects with racialized identity, socioeconomic status, and other social factors to influence health outcomes. Stigma often manifests through implicit biases among medical providers, peers, and even family members, exacerbating health inequities. This review integrates stigma theory with psychophysiological research, emphasizing the need for culturally-sensitive approaches to pain management. By raising awareness of these nuanced dynamics, the authors lay the groundwork to address stigma and its impact on pediatric pain care (Wakefield et al., 2022).

Technological advancements in wearable devices offer new opportunities for monitoring and managing chronic pain in adolescents. The pilot study, “*Feasibility of Wearable Activity Tracking Devices to Measure Physical Activity and Sleep Change Among Adolescents With Chronic Pain,”* evaluates the utility of these devices during a three-week intensive interdisciplinary pain treatment program. The findings reveal significant increases in physical activity and decreased sedentary behavior, demonstrating the potential of wearable devices to track and encourage healthy behaviors. However, challenges in data collection, particularly for sleep metrics, highlight the need for improved device training and engagement strategies, underscoring the promise of integrating personal informatics along with areas requiring further refinement (Junghans-Rutelonis et al., 2024).

Inclusive recruitment practices are essential to ensure diverse representation in pediatric pain research. The study, “*Co-Producing Research Study Recruitment Strategies With and For Children and Young People for Pediatric Chronic Pain Studies,”* takes an innovative approach by involving young patients in designing recruitment strategies. The study identifies barriers to participation through discussions with the Your Rheum advisory group and synthesizes recommendations for improving recruitment. Key suggestions include increasing awareness of research opportunities, leveraging clinical environments for recruitment, and emphasizing the benefits of participation. Researchers can enhance engagement and representation by co-producing strategies with youth, fostering more equitable and impactful studies (Ghio et al., 2024).

The studies in this collection exemplify the breadth of pediatric pain research, its potential to address both psychosocial and biological dimensions of pain, and the critical contributions of women scientists in advancing the field. Together, they highlight the importance of interdisciplinary collaboration and patient-centered approaches, combining psychology, neuroscience, and social science, demonstrating that pain is not merely a physical experience but a multidimensional phenomenon. The *Research Topic* also underscores the need for systemic change to support women in science in addressing pressing health challenges and empowering the next generation of women researchers to lead the way in pediatric pain. As we celebrate the achievements of women in science, let us also recognize the work that remains. By promoting gender equity and dismantling stereotypes, we can ensure that future generations of researchers—regardless of gender—have the opportunity to contribute to the pursuit and dissemination of knowledge. Through these efforts, we can create an equitable and innovative scientific community dedicated to improving the lives of children and adolescents living with pain.
